# Muscle Characteristics and Transcriptomic Analysis of Diploid and Triploid Tiger Pufferfish (*Takifugu rubripes*)

**DOI:** 10.3390/ijms27125210

**Published:** 2026-06-09

**Authors:** Bo Meng, Jingjing Zhang, Shengyu Zhu, Jie Wu, Weidong Li, Haien Zhang, Jianchao Liu, Qian Wang, Changwei Shao

**Affiliations:** 1 College of Fisheries and Life Science, Shanghai Ocean University, Shanghai 201306, China; mengbo2510@163.com (B.M.); jingjingzhang0307@163.com (J.Z.); zhushengyu0703@163.com (S.Z.); 13686302635@163.com (J.W.); 2 State Key Laboratory of Mariculture Biobreeding and Sustainable Goods, Yellow Sea Fisheries Research Institute, Chinese Academy of Fishery Sciences, Qingdao 266071, China; 3 Laboratory for Marine Fisheries Science and Food Production Processes, Laoshan Laboratory, Qingdao 266237, China; 4 Laboratory of Marine Economic Fish Breeding Technology (Ministry of Agriculture and Rural Affairs), Tangshan 063506, China; weidong1970@hotmail.com (W.L.); 18804895712@163.com (H.Z.); guyapiaoyi@163.com (J.L.); 5 Hebei Key Laboratory of the Bohai Sea Fish Germplasm Resources Conservation and Utilization, Beidaihe Central Experiment Station, Chinese Academy of Fishery Sciences, Qinhuangdao 066100, China

**Keywords:** *Takifugu rubripes*, triploid, nutritional composition, hormone levels, transcriptomics

## Abstract

Sexual maturity in tiger pufferfish (*Takifugu rubripes*) consumes substantial metabolic energy, constraining somatic growth and limiting meat yield. Artificial triploid induction (sterility) may redirect energy toward somatic growth. Cold-shock induced triploids were compared with diploid controls using muscle and liver tissues, and the phenotype, histology, nutritional composition, hormone levels, as well as transcriptome profiles were assessed. After 5 months, triploids attained significantly greater body length and body weight, with larger muscle fiber diameter but lower fiber density. The triploids yielded lower ash content and higher amounts of total (TAA), essential (EAA), non-essential (NEAA), and delicious amino acids (DAA), as well as higher total fatty acids (TFA), total polyunsaturated fatty acids (PUFA), and omega 3 polyunsaturated fatty acids (n-3 PUFA). Growth-related hormones were elevated, including growth hormone (GH), testosterone (T), triiodothyronine (T_3_), and thyroxine (T_4_). Muscle transcriptome sequencing identified 231 differentially expressed genes, predominantly enriched in pathways related to cell growth regulation, metabolic processes, and energy conversion. These results provide reference data for breeding programs.

## 1. Introduction

The tiger pufferfish (*Takifugu rubripes*) is a commercially important species native to the northwestern Pacific, including the Sea of Japan, East China Sea, and Yellow Sea. Juveniles exhibit broad salinity tolerance and can inhabit estuarine environments. Owing to its compact genome, *T. rubripes* has become a widely used model in comparative vertebrate genomics [1]. With advances in artificial breeding and industrialized farming, *T. rubripes* aquaculture has expanded in recent years and is increasingly shifting toward large-scale production [2]. However, once fish enter sexual maturation, substantial energy can be diverted to gonadal development, potentially slowing somatic growth and compromising flesh quality, thereby representing a major constraint on production efficiency [3].

Induced triploidy is a practical strategy to reduce reproductive investment by generating fish with reduced fertility or functional sterility. In principle, triploidy can mitigate maturation-associated trade-offs by reallocating resources toward somatic growth, potentially improving production efficiency and stabilizing flesh quality during the reproductive season [4]. Similar strategies have been implemented in many aquaculture species. For example, triploid rainbow trout (*Oncorhynchus mykiss*) has been adopted in salmonid farming to reduce maturation-associated impacts on growth and fillet quality. They can also influence muscle development and flesh quality, exhibiting fewer muscle fibers per myotome but larger fiber size, resulting in a compensatory hypertrophy [5,6]. Triploidy is furthermore associated with altered endocrine profiles. In triploid crucian carp, genes in the growth hormone/insulin-like growth factor (GH/IGF) axis are upregulated, which is hypothesized to contribute to faster growth [7]. These findings revealed that the effects of triploidy on growth performance, muscle traits, nutritional composition, and hormonal regulation can vary among species, highlighting the importance of species-specific studies.

Skeletal muscle represents a key economic trait in *T. rubripes*, valued for its fine texture, desirable taste, and high nutritional value, which underpin strong market demand. In aquatic foods, muscle quality is a primary determinant of consumer acceptance [8]. Accordingly, rigorous evaluation of muscle quality typically integrates both nutritional composition and sensory-related attributes [9]. Fish muscle is an important source of high-quality protein [10] and is notably rich in unsaturated fatty acids [11]. Moreover, the sensory profile of fish muscle is shaped by the interplay among free amino acids and umami- and flavor amino acids, which together modulate overall palatability [12,13].

With the rapid development of transcriptomic approaches, our understanding of the molecular and regulatory architectures underlying physiological processes has deepened substantially. In aquaculture research, transcriptomics has been widely adopted to investigate key traits such as fish growth and development [14] as well as nutritional regulation [15]. For instance, a comparative muscle transcriptome analysis of full-sib *Erythroculter ilishaeformis* individuals exhibiting pronounced growth divergence, it was reported that differentially expressed genes (DEGs) were predominantly enriched in pathways related to protein biosynthesis, digestion, and RNA transport [16].

Here, we compared skeletal muscle from diploid and triploid *T. rubripes* by integrating histological examination, nutritional composition profiling and RNA-seq. Our objectives were to compare the nutritional and flavor-related attributes between diploids and triploids, and identify core genes and signaling pathways that may mediate triploidy-associated regulation of muscle growth and nutrient deposition. This work provides a mechanistic basis to support the genetic improvement of *T. rubripes*, thereby contributing to the long-term sustainability of aquaculture production.

## 2. Results

### 2.1. Growth Performance of Triploid and Diploid T. rubripes

**Figure 1:**
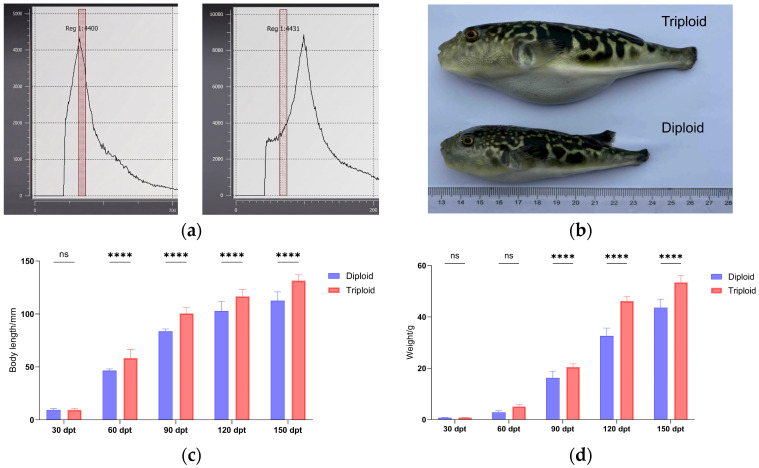
(**a**) Ploidy verification by flow cytometry. The fluorescence intensity of triploid cells (right) is 1.5 times that of diploid control cells (left) in 1-month-old fish. (**b**) Morphological comparison showing the significant somatic growth advantage of triploids compared to diploids in 5-month-old fish. Statistical analysis of body length (**c**) and weight (**d**) between diploid and triploid *T. rubripes*. (****, *p* < 0.0001; ns, *p* ≥ 0.05).

Ploidy status was first validated by flow cytometry, which confirmed that triploid individuals harbored ~1.5-fold higher DNA content than diploid controls (Figure 1a). By 5 months of age, triploids displayed a clear advantage in somatic growth (Figure 1b). As shown in Figure 1c,d, triploid *T. rubripes* demonstrated significantly elevated growth performance than diploids. The body length of triploids was significantly increased from 60 days post-treatment (dpt) onward (Figure 1c, *p* < 0.001), and the body weight of triploids became significantly higher from 90 dpt onward (Figure 1d, *p* < 0.001).

### 2.2. Histological Observations and Morphometrics of Muscle Fibers

**Figure 2:**
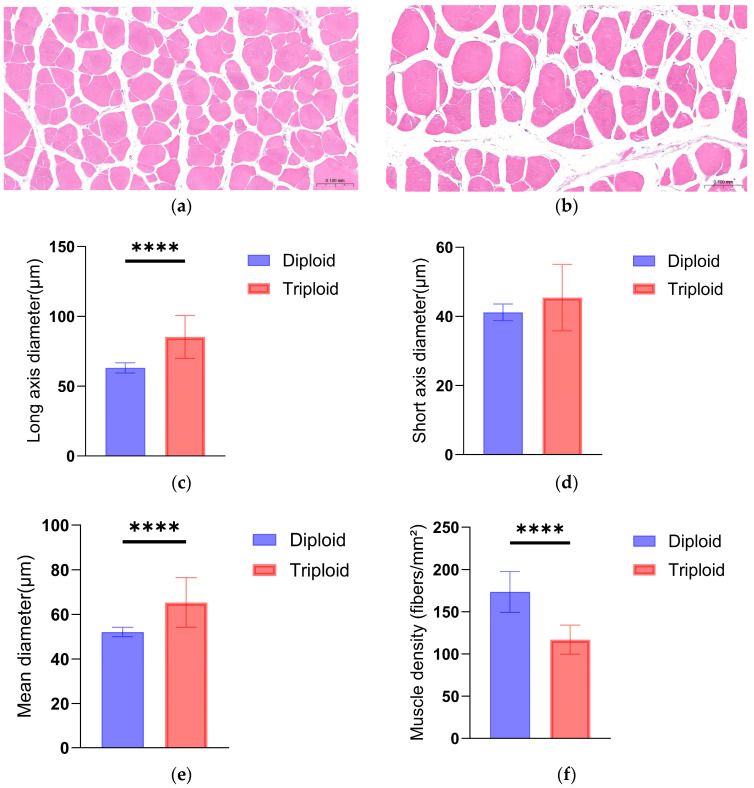
Histological characterization and morphometric quantification of skeletal muscle fibers in 5-month-old diploid and triploid *T. rubripes*. (**a**,**b**) Representative hematoxylin and eosin (H&E)-stained transverse sections showing myofiber architecture in (**a**) diploid and (**b**) triploid groups. Scale bar = 100 μm. (**c**–**f**) Quantification of myofiber morphometrics: (**c**) long axis diameter, (**d**) short axis diameter, (**e**) mean diameter, and (**f**) myofiber density (fibers/mm^2^). Data are presented as mean ± SD. **** *p* < 0.0001; unmarked comparisons are not significant.

Histological analysis revealed that the myofibers of both diploid and triploid *T. rubripes* exhibited irregular polygonal cross-sections (Figure 2a,b). Diploid muscle was characterized by finer, uniformly distributed fibers with small interstitial spacing, resulting in a compact, high-density organization (Figure 2a). In contrast, triploids exhibited enlarged fibers separated by wider interstitial gaps, leading to a looser structural arrangement and objectively lower fiber density (Figure 2b).

Quantitative analysis of dorsal muscle cross-sections (Figure 2c–f) confirmed these observations. Triploids exhibited significantly greater muscle fiber long axis diameters and mean diameters compared to diploids (Figure 2c, *p* < 0.0001). The short diameter was also larger in triploids, although the difference was not statistically significant (Figure 2d). Consequently, the mean myofiber diameters in triploids were significantly greater than those in diploids (Figure 2e, *p* < 0.0001). In addition, myofiber density in triploids was significantly lower than that in diploids (Figure 2f, *p* < 0.0001).

### 2.3. Muscle Nutritional Composition

Proximate composition analysis showed that moisture, crude protein, crude lipid, and total sugars did not differ significantly between diploid and triploid *T. rubripes* (Table 1, *p* > 0.05). In contrast, ash content—an indicator of total mineral content—was significantly higher in diploids than in triploids (Table 1, *p* < 0.05).

Amino acid profiling indicated that triploid muscle contained significantly higher total amino acids (TAA), essential amino acids (EAA), non-essential amino acids (NEAA), and delicious amino acids (DAA) than diploid muscle (Table 2, *p* < 0.05). The DAA/TAA ratio was also significantly elevated in triploids (Table 2, *p* < 0.05), while EAA/TAA and EAA/NEAA ratios did not differ between groups (Table 2, *p* > 0.05).

Analysis of fatty acid profiles (Table 3) showed that total fatty acids (TFA) were marginally but significantly higher in triploids than in diploids (*p* < 0.05). Saturated fatty acids (SFA) and monounsaturated fatty acids (MUFA) did not differ between diploid and triploid groups (*p* > 0.05), whereas polyunsaturated fatty acids (PUFA) were marginally but significantly higher in triploid muscle (*p* < 0.05). This difference was primarily attributable to the n-3 PUFA fraction, which was elevated in triploids (*p* < 0.05), while n-6 PUFA and the n-3/n-6 ratios were comparable between groups (*p* > 0.05). In addition, triploids displayed marginally higher PUFA/SFA and DHA/EPA ratios than diploids (*p* < 0.05), consistent with a more favorable fatty-acid profile (Table 3).

### 2.4. Endocrine Characterization

Analysis of liver tissue hormone profiles revealed that triploids had significantly higher concentrations of growth hormone (GH), testosterone (T), triiodothyronine (T_3_) and thyroxine (T_4_) than diploids (*p* < 0.05) (Table 4).

### 2.5. Transcriptomic Analysis

#### 2.5.1. Transcriptome Sequencing Results

**Figure 3:**
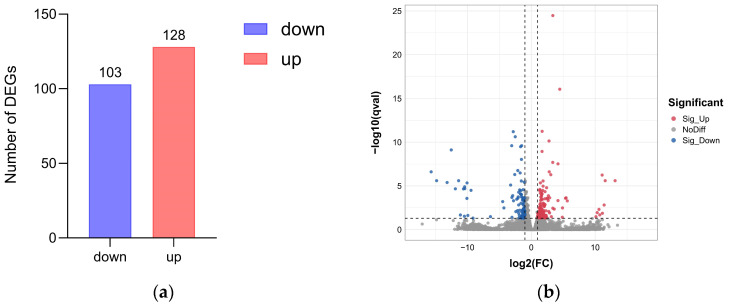
Muscle transcriptome analysis of diploid and triploid *T. rubripes*. (**a**) Histogram showing the number of down- and up- regulated differentially expressed genes (DEGs) in triploids relative to diploids. (**b**) Volcano plot showing differentially expressed genes of triploids vs. diploids comparison in the muscles. The horizontal dashed line represents the significance threshold (*q*-value < 0.05), and the vertical dashed lines represent the fold change thresholds (|log2 fold change| ≥ 1).

The diploid group and triploid group were sampled to investigate the transcriptomic alterations associated with ploidy-dependent muscle development. Each group comprised three biological replicates. Sequencing quality metrics were summarized in Table 5. Across samples, raw reads ranged from 42,418,432 to 48,298,894, with 40,845,270 to 46,726,528 clean reads retained after filtering. Base quality was consistently high (Q20, 100%; Q30, 98.92–99.02%), and GC content was stable (52.0–52.5%), indicating robust sequencing performance and consistency among samples. Overall alignment rates to the reference genome were high (96.43–96.98%), supporting the reliability of downstream expression quantification and differential expression analyses. Statistical analysis identified 231 DEGs between triploid and diploid *T. rubripes*. Relative to diploids, triploids showed 103 downregulated and 128 upregulated genes (Figure 3).

#### 2.5.2. GO and KEGG Enrichment Analysis

**Figure 4:**
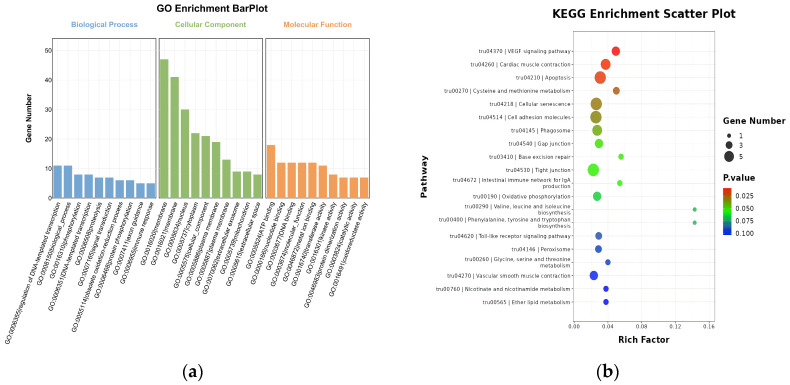
GO and KEGG enrichment analyses of DEGs in triploids relative to diploids identified in muscle. (**a**) GO enrichment of DEGs in the Biological Process (BP), Cellular Component (CC), and Molecular Function (MF) categories; bar height indicates the number of DEGs annotated to each GO term. (**b**) KEGG pathway enrichment of DEGs; the rich factor is defined as the ratio of DEGs mapped to a pathway to the total number of genes annotated in that pathway. Dot size represents the number of DEGs, and dot color indicates the *p* value (red, lower *p* value/higher significance).

Gene Ontology (GO) enrichment analysis (Figure 4a) showed that differentially expressed genes (DEGs) span multiple functional categories. In the Biological Process (BP) category, DEGs were enriched in regulation of DNA-templated transcription and biological process, alongside keys processes such as phosphorylation and development-related terms. In the Cellular Component (CC) category, DEGs were enriched in membrane-related terms, specifically membrane, followed by nucleus and cytoplasm, which is highly consistent with alterations in cellular architecture induced by triploid. In the Molecular Function (MF) category, adenosine triphosphate (ATP) binding exhibited the highest gene count, followed by nucleotide binding and DNA binding.

KEGG pathway enrichment (Figure 4b) further indicated that DEGs were over-represented in pathways related to signal transduction, cell growth and death, and metabolism. Among all enriched pathways, the top three most statistically significant pathways were Vascular endothelial growth factor (VEGF) signaling pathway, Cardiac muscle contraction, and Apoptosis. Detailed analysis revealed that the high significance of the VEGF signaling pathway directly implicates robust angiogenesis-related remodeling in the muscle tissue of triploids. In addition, Valine, leucine and isoleucine biosynthesis exhibited the highest enrichment factor, indicating divergence in amino-acid metabolism between diploids and triploids. Pathways linked to cellular homeostasis, including Cell adhesion molecules, was also significantly enriched.

#### 2.5.3. RT-qPCR Validation of DEGs

**Figure 5:**
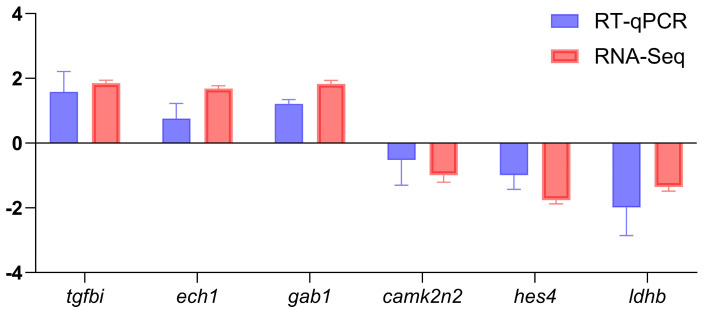
Validation of transcriptomic data by RT-qPCR. Relative expression levels of representative genes were analyzed to verify the RNA-seq results.

To validate the RNA-seq results, RT-qPCR was performed on six DEGs: *gab1*, *hes4*, *camk2n2*, *tgfbi*, *ech1*, and *ldhb*. Expression patterns across groups were concordant with the RNA-seq data (Figure 5), supporting the robustness of the transcriptomic analysis.

## 3. Discussion

This study systematically compared diploid and triploid *T. rubripes* by integrating analyses of muscle histomorphology, proximate composition, endocrine profiles, and transcriptomic profiling. The results show that triploidy is associated with distinct advantages in muscle development and nutritional quality, while integrative analyses identify cellular, endocrine, and molecular pathways associated with this enhanced growth. Collectively, these results outline a mechanistic framework connecting the growth enhancements seen in triploids with myofiber hypertrophy, endocrine activation, nutrient accumulation, and the transcriptional regulation of muscle development.

Across the 150-day growth trial, triploid *T. rubripes* exhibited an obvious growth advantage over diploids, with body weight diverging from 90 dpt onward and total length from 60 dpt onward. The earlier divergence in total length than in body weight may suggest that triploids may preferentially allocate energy to skeletal muscle elongation prior to achieving mass gain in early development [17]. The growth advantage observed in triploid *T. rubripes* may be associated with ‘reproductive energy reallocation’. It is hypothesized that the disruption of meiosis in triploids leads to sterility, theoretically allowing energy originally intended for gonadal development to be redirected toward muscle growth [18,19]. While transcriptomic profiling reveals an enrichment of growth and metabolic pathways, further histological and physiological evaluations of the gonads are required to definitively substantiate the reproductive energy reallocation hypothesis in *T. rubripes*.

Histological analyses showed that triploids had significantly larger muscle-fiber major-axis and mean diameters than diploids, accompanied by reduced fiber density, consistent with a looser muscle architecture. Larger standard deviations in triploids further suggest greater heterogeneity in fiber size relative to the more compact and uniform organization in diploids. Similar trends have been reported for diploid–triploid comparisons in *O. mykiss* [20,21,22]. These differences may arise from altered energy allocation in triploids, with energy that would typically support gonadal development instead promoting somatic growth, leading to larger myofibers and lower fiber density [4]. Previous studies have linked smaller fiber diameters and higher fiber density to improved tenderness [23]. Consequently, the enlarged myofibers observed in triploids may affect flesh texture, though direct texture measurements and sensory evaluations are needed to confirm this.

Muscle quality is a key production trait in *T. rubripes*. Nutritional value cannot be assessed solely from proximate indices such as crude protein; it should also consider amino-acid composition, absolute abundance, and proportional profiles [24]. Accordingly, no significant differences were observed between diploids and triploids in moisture, crude protein, or total sugar content. However, ash content was significantly lower in triploids than in diploids. This reduction may be related to ploidy-dependent differences in mineral accumulation and structural development, which is in line with the growth characteristics described above. In contrast, triploids displayed certain advantages in nutrient composition and flavor-associated components in the present study [25,26]. In terms of amino acids, triploids exhibited higher total amino acids (TAA) and significantly elevated essential amino acids (EAA), non-essential amino acids (NEAA) and delicious amino acids (DAA) compared with diploids. While the EAA/TAA and EAA/NEAA ratios remained consistent between groups, suggesting that triploidy increases the absolute nutrient abundance without altering the fundamental protein quality balance, the significantly elevated EAA content highlights the superior nutritional value of triploid muscle. Furthermore, triploids displayed a significantly higher delicious amino acids (DAA) content and an increased DAA/TAA ratio. Consistently, studies in bream have reported that enrichment of flavor-active amino acids is associated with more desirable taste attributes [27]. Meanwhile, enrichment of flavor-active amino acids may contribute to differences in perceived palatability, although direct sensory evaluation would be required to confirm this inference [28]. Triploids showed a higher proportion of polyunsaturated fatty acids (PUFAs), particularly n-3 PUFAs. Unsaturated fatty acids are widely recognized for their health-promoting effects, including lipid-lowering, antihypertensive, antitumor, and immunomodulatory activities, and may reduce cardiovascular risk [29]. In the study, triploids showed marginally but significantly higher PUFA/SFA and DHA/EPA ratios. Previous studies have demonstrated that EPA and DHA play important roles in promoting brain development and enhancing memory [30,31]. PUFAs can also enhance sensory quality by contributing to desirable aroma and improving juiciness of the flesh [32]. Thus, triploid *T. rubripes* may offer enhanced nutritional value, favorable flavor profiles, and improved health benefits, making them a promising option for aquaculture.

*T. rubripes* triploids displayed distinct endocrine profiles with diploids, including significantly higher levels of growth hormone (GH), testosterone (T), and thyroid hormones (T_4_ and T_3_). These differences are consistent with altered endocrine status that may contribute to enhanced somatic growth. GH plays a central role in fish growth regulation, mainly through the GH/IGF axis, which supports tissue growth by promoting IGF-I production, nutrient utilization, and protein deposition. The importance of this pathway in teleost growth has been widely documented, and ploidy-associated upregulation of GH/IGF-axis genes has also been reported in triploid crucian carp [7,33]. The elevated GH level in triploid *T. rubripes* may indicate stronger growth-related endocrine signaling, which could promote muscle accretion and contribute to increased body mass. In parallel, thyroid hormones play key roles in metabolic activity, energy turnover, and nutrient utilization [34,35]. The elevated T_4_ and T_3_ levels in triploids may indicate a higher metabolic state, potentially enhancing energy availability and substrate turnover to support rapid somatic growth. Increased testosterone levels further suggest broader endocrine differences between triploids and diploids, although its specific role in muscle growth remains unclear. Collectively, these endocrine changes indicate that hormonal regulation, particularly through the GH/IGF axis and thyroid hormone-mediated metabolism, may contribute to the growth advantage observed in triploid *T. rubripes*.

Transcriptomic profiling revealed that the molecular divergence between diploid and triploid *T. rubripes* muscle encompasses multiple functional themes related to transcriptional regulation, structural remodeling, and energy metabolism. Several transcription-related DEGs, including *LOC101068176*, *zbtb10* and *LOC115248360*, were upregulated in triploid muscle. Consistently, GO enrichment analysis highlighted transcription-related processes within the Biological Process (BP) category, suggesting that ploidy-associated differences in muscle development are closely linked to gene regulatory reprogramming [36,37]. Within the Cellular Component and Molecular Function categories, genes associated with contractile activity, cytoskeletal organization, and nucleotide binding showed triploid-biased expression, further supporting structural remodeling of muscle fibers [38,39]. Notably, *LOC105417828*, *diaph2* and *LOC101061790* were linked to membrane and cytoplasm—consistent with proposed molecular mechanisms underlying the “cell hypertrophy” phenotype reported in zebrafish [36]. The differential expression of ATP binding and nucleotide binding genes, including *atp8a1* and *atp11c*, further indicates active nucleotide turnover and energy-dependent cytoskeletal remodeling [40,41,42]. Previous studies showed that such energetic reinforcement may sustain the elevated demands of rapid growth and cytoskeletal reorganization, thereby providing a mechanistic explanation for the growth advantage observed in triploids during the growth trial [43].

KEGG pathway analysis further elucidated physiological adaptations that may enable triploids to sustain rapid growth while maintaining superior flesh quality. The significant enrichment of the VEGF signaling pathway in the dataset suggests that vascular and hypoxia-related adaptation may contribute to triploid muscle development. This enrichment was reflected by the differential expression of *shc2* and *LOC101064045*, together with vascular- or hypoxia-associated genes such as *higd1a*, *tmem100*, *cxcl12* and *calcrl*. These transcriptional changes may help adjust oxygen and nutrient supply in triploid muscle, thereby supporting the higher metabolic demand associated with marked myofiber hypertrophy. This interpretation is consistent with previous studies showing that VEGF activity promotes vascular network expansion and supports tissue growth under increased metabolic requirements [44,45]. In addition, DEGs enriched in Cardiac muscle contraction and Cell adhesion molecules—including *LOC101077666*, *LOC101067100* and *LOC101065280*—suggested that triploidy is associated with remodeling of mitochondrial energy metabolism, cytoskeletal architecture, and intercellular adhesion. These processes may, in turn, contribute to texture-related changes in triploid muscle [46]. Enrichment of the Cysteine and methionine metabolism pathway, represented by *LOC101075429*, *tat* and *ldhb*, suggests ploidy-associated reshaping of amino acid metabolism in triploid muscle. This metabolic adjustment is consistent with the observed amino acid accumulation and may contribute to the improved nutritional profile of triploid muscle [47]. Finally, the Apoptosis and Cellular senescence, along with the involvement of genes such as *parp3*, *ctsh* and LOC101067560, indicates that triploid muscle may sustain cellular homeostasis during accelerated growth through coordinated regulation of cell survival, stress responses, and senescence-associated processes [48,49,50,51].

In the RNA-seq validation, we selected six genes (*gab1*, *hes4*, *ech1*, *ldhb*, *tgfbi*, and *camk2n2*). qPCR expression patterns not only supported the reliability of the transcriptomic dataset, but also provided additional insight into gene-level differences associated with muscle phenotypes in triploids. Specifically, *gab1*, which is associated with muscle cell proliferation and differentiation, was upregulated in triploid muscle, while *hes4*, a gene involved in muscle stem cell regulation and differentiation, was downregulated [52,53,54]. This suggests that triploids adopt a specific “myofiber hypertrophy and differentiation” regulatory strategy, providing molecular evidence for the cell gigantism phenomenon observed in histological sections. Furthermore, *ech1*, involved in fatty acid β-oxidation, and *ldhb*, which catalyzes the conversion of lactate to pyruvate (aerobic metabolism), exhibited differential expression [55,56]. This reflects adaptive adjustments in mitochondrial fatty acid β-oxidation and lactate clearance, which closely align with the changes in the fatty acid profile of muscle nutritional composition observed in this study. Research has shown that *tgfbi*, a secreted protein, may influence collagen deposition and muscle fibrosis, while *camk2n2*, an endogenous inhibitor of CaMKII, regulates calcium signaling in muscles [57,58]. The differential expression of *tgfbi* and *camk2n2* further suggests that cell enlargement in triploid *T. rubripes* muscle may be accompanied by coordinated regulatory changes in extracellular matrix remodeling and calcium signaling pathways. Collectively, these gene-level changes highlight coordinated differences across growth-related signaling, energy metabolism, and structural remodeling in triploid *T. rubripes* muscle.

In summary, this study revealed that triploid *T. rubripes* exhibited improved growth performance and selected nutritional indices relative to diploids. Phenotypically, triploids displayed accelerated weight gain accompanied by pronounced myofiber hypertrophy; physiologically, these traits were associated with endocrine differences, including elevated GH and thyroid hormone levels; and nutritionally, triploid muscle was relatively enriched in essential amino acids, flavor-active amino acids, and n-3 polyunsaturated fatty acids. Transcriptomic profiling further suggests that triploid divergence involves pathways related to angiogenesis, structural remodeling, and energy metabolism. Collectively, these findings provide multi-level evidence that spanning tissue architecture, endocrine profiles, and molecular signatures to support candidate mechanisms underlying enhanced growth in triploid fish and providing a basis for broodstock improvement in *T. rubripes* aquaculture.

## 4. Materials and Methods

### 4.1. Experimental Materials and Sample Collection

Five-month-old diploid and triploid *T. rubripes* were used in this study. Triploid fish were produced from diploid fertilized eggs by a cold-shock protocol: embryos were exposed to 2 °C starting 5 min post-fertilization for 15 min [59], and triploidy was confirmed by flow-cytometric analysis of DNA content using a CyFlow^®^ Ploidy Analyser (Sysmex Partec GmbH, Goerlitz, Germany). All juveniles were reared at the aquaculture facility of Haidu Fishery & Food Co., Ltd. (Tangshan, Hebei, China) in 3 m^3^ flow-through tanks under identical conditions, with water temperature maintained at approximately 19–20 °C and salinity at ~30‰. Fish were fed a commercial pellet diet (50.0% crude protein and 8.0% crude lipid) at a daily ration equivalent to ~5% of body weight. At 5 months post-fertilization, fish were fasted for 24 h and anesthetized with Tricaine methanesulfonate (MS-222; Sigma, Shanghai, China) before sampling. In each ploidy group, three fish were randomly selected for each downstream assay: dorsal trunk muscle was dissected and split, with one portion fixed in 4% paraformaldehyde (PFA, Solarbio, Beijing, China) for histological analysis and the remaining portion frozen in liquid nitrogen and stored at −80 °C for transcriptome sequencing, RT-qPCR validation, and proximate/nutritional composition analysis. Liver tissues were sampled, frozen in liquid nitrogen, and stored at −80 °C for hormone quantification.

### 4.2. Histological Analysis

Muscle tissues were fixed in PFA for at least 24 h, then dehydrated through a graded ethanol series (Sinopharm Chemical Reagent Co., Beijing, China), cleared in xylene (BaSO, Guangzhou, China), and embedded in paraffin wax (Leica, Wetzlar, Germany). Paraffin blocks were sectioned at 5 μm thickness using a rotary microtome (Leica RM2016, Leica Microsystems, Wetzlar, Germany). Sections were stained with hematoxylin (BaSO, Guangzhou, China) and eosin (Sinopharm Chemical Reagent Co., Beijing, China) following standard protocols, then examined under a light microscope. Representative images were captured with a digital camera attached to the microscope. Muscle fiber diameters and densities were quantified from micrographs using Image-Pro Plus software (Media Cybernetics, version 6.0).

### 4.3. Proximate Composition Analysis

The proximate composition of *T. rubripes* muscle was determined following standard methods recommended by the Association of Official Analytical Chemists [60]. Briefly, moisture content was measured by drying the sample at 105 °C to constant weight. Ash content was determined by placing the sample in a porcelain crucible, pre-carbonizing it on a hot plate until completely charred (with no visible smoke), and then incinerating it at 550 °C for 4 h in a muffle furnace (SX2-12-10, Longkou Electric Furnace Manufacturing Plant, Longkou, China). Crude protein content was measured using the Kjeldahl method; total nitrogen was converted to protein using a conversion factor of 6.25 (N × 6.25), and analyses were performed using a Kjeldahl nitrogen analyzer (K9840, Jinan Haineng Instruments Co., Jinan, China). Crude fat content was determined by acid hydrolysis: lipids released after hydrolysis with hydrochloric acid (Sinopharm Chemical Reagent Co., Beijing, China) were extracted with anhydrous diethyl ether (Laiyang Kangde Chemical Co., Laiyang, China) and quantified gravimetrically. Total carbohydrates (total sugars) were determined using the phenol–sulfuric acid colorimetric method with D-glucose as the calibration standard; absorbance was measured at 490 nm using a UV–Vis spectrophotometer (A8453, Agilent Technologies, Santa Clara, CA, USA), and the content was calculated accordingly.

### 4.4. Determination of Amino Acid Composition

Muscle samples were hydrolyzed with 6 M HCl containing trace phenol in sealed tubes at 110 °C for 22 h, following three cycles of freezing, evacuation, and nitrogen flushing. The hydrolysates were cooled, filtered, and diluted to a final volume of 50 mL. A 1.0 mL aliquot was evaporated to dryness under reduced pressure at 50 °C, with repeated water addition to remove residual acid. The dried residue was dissolved in sodium citrate buffer (pH 2.2) (Sinopharm Chemical Reagent Co., Ltd., Beijing, China) and filtered through a 0.22 μm membrane. Amino acids were analyzed using an automatic amino acid analyzer (LA8080, Hitachi, Tokyo, Japan) with detection at 570 and 440 nm. Standards and samples were injected at equal volumes, and amino acid concentrations were quantified by external standardization based on peak areas.

### 4.5. Determination of Fatty Acid Composition

Muscle samples were hydrolyzed with 8.3 M HCl at 80 °C for 40 min, followed by three extractions with diethyl ether–petroleum ether (1:1, *v*/*v*) (Sinopharm Chemical Reagent Co., Beijing, China). The combined extracts were concentrated to dryness using a rotary evaporator (RE-2000B; Yarong Biochemical Instrument Factory, Shanghai, China). The recovered lipids were sequentially saponified with 2% NaOH in methanol (Tianjin Beilian Fine Chemical Development Co., Tianjin, China) and methylated with 15% boron trifluoride (BF_3_) in methanol (CNW, Shanghai, China) under reflux at 80 °C to generate fatty acid methyl esters (FAMEs). The reaction products were extracted with n-heptane, dried over anhydrous sodium sulfate, and filtered through a 0.22-μm membrane. FAMEs were analyzed by capillary gas chromatography on a 7890A system (Agilent Technologies, Santa Clara, CA, USA) equipped with a 100 m × 0.25 mm i.d. capillary column (0.20 μm film thickness). The oven temperature program was: 100 °C for 13 min; ramp to 180 °C at 10 °C/min and hold for 6 min; ramp to 192 °C at 1 °C/min and hold for 9 min; ramp to 230 °C at 3 °C/min and hold for 6 min. Injector and detector temperatures were both set to 240 °C, and nitrogen was used as the carrier gas. Samples were injected at a split ratio of 20:1, and fatty acids were quantified by peak area normalization.

### 4.6. Determination of Hormone Levels in Liver Tissue

Growth hormone (GH), testosterone (T), thyroxine (T_4_), and triiodothyronine (T_3_) were quantified in *T. rubripes* liver tissue using enzyme-linked immunosorbent assay (ELISA) kits (Shanghai Yuanju Biotechnology Center, Shanghai, China) according to the manufacturer’s instructions. Absorbance was measured at 450 nm using a microplate reader, and hormone concentrations were calculated from standard curves. Intra-assay and inter-assay coefficients of variation were both <15%.

### 4.7. Transcriptome Sequencing and Data Analysis

Total RNA was isolated and purified from skeletal muscle samples of diploid and triploid *T. rubripes* using TRIzol reagent according to the manufacturer’s protocol, with three biological replicates per group (NC_1-3 and Tri_1-3, respectively). Sequencing libraries were prepared using standard protocols and sequenced on an Illumina NovaSeq 6000 platform (Illumina, Tokyo, Japan).

Raw reads were processed with fastp to remove adapter sequences and low-quality reads. Clean reads were aligned to the *T. rubripes* reference genome (reference genome: fTakRub1.2, RefSeq GCF_901000725.2) using HISAT2. Gene expression was quantified and normalized as transcripts per million (TPM). Differentially expressed genes (DEGs) were defined using the following criteria of |log2 fold change| ≥ 1 and *q* < 0.05 [61]. Identified DEGs were used for downstream functional enrichment analyses.

### 4.8. Evaluation of Gene Expression by RT-qPCR

To validate the reliability of the RNA-seq data, six genes (*gab1*, *hes4*, *ech1*, *ldhb*, *tgfbi,* and *camk2n2*) were selected for RT-qPCR analysis (Table A1). Gene-specific primers were designed using Primer Premier 5.0 and synthesized by RiboBio Company, Guangzhou, China. RT–qPCR was performed using the QuantiNova SYBR Green PCR Kit (Qiagen, Hilden, Germany) on a LightCycler^®^ 480 II Real-Time PCR System (Roche, Basel, Switzerland), following the manufacturer’s protocol. Thermal cycling was conducted with an initial denaturation at 95 °C for 2 min, followed by 40 cycles of 95 °C for 5 s and 60 °C for 10 s. Melt-curve analysis was performed by holding at 95 °C for 15 s and 60 °C for 1 min, followed by a ramp to 95 °C at 1 °C/min and a final hold for 15 s. The *18S rRNA* served as the internal reference gene. All reactions were performed in triplicate (technical replicates). Relative expression levels were calculated using the 2^−ΔΔCt^ method.

### 4.9. Statistical Analysis

Statistical analyses were performed using GraphPad Prism 10.1.2 software (GraphPad Software, San Diego, CA, USA). Data are presented as mean ± standard deviation (SD). Differences between groups were analyzed using Student’s *t*-test, and *p* < 0.05 was considered statistically significant.

## Figures and Tables

**Table 1:** Proximate composition of each group (per 100 g fresh muscle weight, %).

Nutrients	Diploid	Triploid
Moisture	79.46 ± 0.06	79.44 ± 0.09
Crude protein	17.48 ± 0.10	17.36 ± 0.07
Crude lipid	0.92 ± 0.05	1.04 ± 0.08
Ash	1.03 ± 0.06 ^a^	0.74 ± 0.01 ^b^
Total sugar	0.61 ± 0.03	0.60 ± 0.01

Note: Values are presented as mean ± SD. Different lowercase superscript letters within the same row indicate significant differences between groups (*p* < 0.05).

**Table 2:** Amino acid composition of each group (per 100 g fresh muscle weight, %).

Parameter	Diploid	Triploid
TAA	13.64 ± 0.21 ^a^	15.10 ± 0.13 ^b^
EAA	5.83 ± 0.08 ^a^	6.39 ± 0.06 ^b^
NEAA	7.82 ± 0.14 ^a^	8.71 ± 0.07 ^b^
DAA	4.81 ± 0.10 ^a^	5.43 ± 0.04 ^b^
EAA/TAA (%)	42.72 ± 0.32	42.34 ± 0.01
EAA/NEAA (%)	74.58 ± 0.97	73.44 ± 0.04
DAA/TAA (%)	35.26 ± 0.34 ^a^	35.94 ± 0.08 ^b^

Note: TAA, total amino acids; EAA, essential amino acids; NEAA, non-essential amino acids; DAA, delicious amino acids (Asp, Glu, Gly, and Ala). Values are presented as mean ± SD. Different lowercase superscript letters within the same row indicate significant differences between groups (*p* < 0.05).

**Table 3:** Fatty acid composition (per 100 g fresh muscle weight, %).

Parameter	Diploid	Triploid
TFA	0.6605 ± 0.0175 ^a^	0.6954 ± 0.0061 ^b^
SFA	0.2465 ± 0.0068	0.2494 ± 0.0023
MUFA	0.1292 ± 0.0033	0.1321 ± 0.0014
PUFA	0.2849 ± 0.0075 ^a^	0.3138 ± 0.0025 ^b^
n-3 PUFA	0.1862 ± 0.0051 ^a^	0.2061 ± 0.0010 ^b^
n-6 PUFA	0.0927 ± 0.0021	0.1009 ± 0.0014
n-3/n-6	2.0090 ± 0.0096	2.0423 ± 0.0175
PUFA/SFA	1.1560 ± 0.0032 ^a^	1.2582 ± 0.0033 ^b^
DHA/EPA	3.7360 ± 0.0051 ^a^	4.0482 ± 0.0513 ^b^

Note: TFA, total fatty acids; SFA, saturated fatty acids; MUFA, monounsaturated fatty acids; PUFA, polyunsaturated fatty acids; n-3 PUFA, omega 3 polyunsaturated fatty acids; n-6 PUFA, omega 6 polyunsaturated fatty acids. Values are presented as mean ± SD. Different lowercase superscript letters within the same row indicate significant differences between groups (*p* < 0.05).

**Table 4:** Hormone contents.

Parameter	Unit	Diploid	Triploid
Growth hormone (GH)	μg/g	3.14 ± 0.10 ^a^	3.94 ± 0.06 ^b^
Testosterone (T)	nmol/g	1.61 ± 0.07 ^a^	2.08 ± 0.08 ^b^
Triiodothyronine (T_3_)	pmol/g	7.84 ± 0.38 ^a^	9.14 ± 0.25 ^b^
Thyroxine (T_4_)	pmol/g	367.50 ± 14.04 ^a^	455.91 ± 6.61 ^b^

Note: Different lowercase superscript letters within the same row indicate significant differences between groups (*p* < 0.05).

**Table 5:** Summary of transcriptome sequencing.

Samples Name	Raw Reads	Clean Reads	Q20 (%)	Q30 (%)	GC Content (%)	Mapping Ratio (%)
NC_1	48,298,894	46,726,528	100	99.02	52.5	96.98
NC_2	45,756,466	44,126,422	100	98.95	52	96.59
NC_3	44,043,950	42,364,912	100	98.92	52	96.43
Tri_1	48,146,282	46,351,312	100	98.97	52	96.69
Tri_2	43,853,188	42,314,676	100	99.01	52	96.70
Tri_3	42,418,432	40,845,270	100	98.96	52	96.64

## Data Availability

The data are contained within the article. RNA-seq data are submitted in NCBI (BioProject: PRJNA1433062).

## Appendix A

**Table A1:** Primers and probes for gene expression analysis (qRT-PCR).

Genes	Sequences (5′-3′)	Product Length	Tm
*ldhb*-F	GACTCAGCTCGCTTCCGTTA	231 bp	60 °C
*ldhb*-R	GATCACCTCGTAGGCACTGT		
*ech1*-F	GGTGATAGAGCGGTGTCCAAA	300 bp	60 °C
*ech1*-R	ATCATGGCCTCCTTATCGGC		
*hes4*-F	GCCAGTGAGCACAGAAAATCAT	258 bp	60 °C
*hes4*-R	CTCGTTGAATCCGGCTCTGT		
*gab1*-F	AGTTGAGACGTTACGCATGGA	296 bp	60 °C
*gab1*-R	GTTGAAGCCGCAGATGTCAC		
*tgfbi*-F	CCGAAGGCTCGTCTGTTACC	276 bp	60 °C
*tgfbi*-R	TCTCGATGCAAAGGTTGTTTCTG		
*camk2n2*-F	TCTGAGGTGCTGCCATACAG	276 bp	60 °C
*camk2n2*-R	CTTCGATGACCACTCGCTTG		
*18S rRNA*-F	GGGCCTTTTATTGGGGGACA	250 bp	60 °C
*18S rRNA*-R	CGCTTTCATGGCCTGTGTTG		
